# Mechanical and histological evaluation of a titanium device for orthodontic anchorage, placed with or without cyanoacrylate adhesive

**DOI:** 10.1590/2177-6709.24.3.071-078.oar

**Published:** 2019

**Authors:** Anderson Antonio Mamede, Elizabeth Ferreira Martinez, Roberta Tarkany Basting

**Affiliations:** 1 Instituto e Centro de Pesquisas São Leopoldo Mandic (Campinas/SP, Brazil).

**Keywords:** Cortical bone, Orthodontic anchorage procedures, Fractures

## Abstract

**Objective:**

The objective of the present study was to perform a histological evaluation of a titanium mini-implant for orthodontic anchorage. Shear strength and fracture patterns that occurred immediately, 30 and 60 days after insertion with or without N-2-butyl-cyanoacrylate adhesive were evaluated.

**Methods:**

Ninety-six mini-implants (Arrow, Peclab, Brazil) were placed in the tibia of 9 male rabbits, with or without an adhesive (Vetbond™, 3M, USA). Histological evaluation was done by optical light microscope. Shear strength testing was performed, followed by fracture analysis with visual inspection.

**Results:**

Close contact between the newly formed bone and the device was evidenced in the group without adhesive, whereas gaps in the group with adhesive were found. Tukey test showed similar values in both groups at the immediate time point (20.70 N without adhesive and 24.69 N with adhesive), and higher values for the non-adhesive group, after 30 and 60 days (43.98 N and 78.55 N, respectively). The values for the adhesive group were similar for the immediate time point (24.69 N), 30 days (18.23 N) and 60 days (31.98 N). The fractures were adhesive for both groups at the immediate time point. The fractures were cohesive in bone for the non-adhesive group after 30 and 60 days.

**Conclusions:**

The mini-implants showed close bone contact and required higher shear strength for removal at 30 and 60 days for the non-adhesive group. Further studies are needed to assess the proper way to remove the orthodontic anchorage without cohesive fractures in bone.

## RESUMO

**Objetivos:**

este estudo teve como objetivo realizar uma avaliação histológica de um mini-implante para ancoragem em Ortodontia. Avaliou-se, também, a carga de cisalhamento e o padrão de fratura imediatamente e após 30 e 60 dias da sua inserção, com ou sem o uso do adesivo N-butil-2-cianoacrilato.

**Métodos:**

noventa e seis mini-implantes (Arrow, Peclab, Brasil) foram instalados na tíbia de nove coelhos machos, com ou sem adesivo (Vetbond™, 3M, EUA). A avaliação histológica foi realizada com uso de microscópico de luz óptica. Realizou-se o teste de resistência ao cisalhamento, seguido pela análise da fratura, por meio de inspeção visual.

**Resultados:**

um contato íntimo entre o novo osso formado e o dispositivo foi evidenciado no grupo sem adesivo, enquanto espaços foram encontrados no grupo com adesivo. O teste de Tukey mostrou valores semelhantes em ambos os grupos no tempo imediato (20,70 N sem adesivo e 24,69 N com adesivo), e valores maiores para o grupo sem adesivo após 30 e 60 dias (43,98 N e 78,55 N, respectivamente). Os valores para o grupo com adesivo foram semelhantes para os tempos imediato (24,69 N), 30 dias (18,23 N) e 60 dias (31,98 N). As fraturas foram adesivas para ambos os grupos, no tempo imediato. As fraturas foram coesivas no osso para os grupos sem adesivo, após 30 e 60 dias.

**Conclusões:** os mini-implantes mostraram um contato íntimo com o osso e requereram alta carga de cisalhamento para sua remoção após 30 e 60 dias nos grupos sem adesivo. Estudos adicionais são necessários para avaliar um método para remoção do dispositivo ortodôntico sem fratura coesiva no osso.

## INTRODUCTION

The search for stable anchorage in orthodontics has been sought over the years, particularly because of low compliance by patients. Traditional extra- and intraoral devices are being replaced by osseointegrated implants, including palatal implants, onplants, miniplates and mini-implants, in an effort to achieve higher success rates and shorter treatment time.^1^ Despite the clinical advantages of mini-implants, there are some disadvantages, such as risk of root damage, nasal floor or maxillary sinus perforation,^2^ tooth ankylosis,^3^ and lack of space for insertion, among others.^1^

Seeking a simple and safer way to achieve skeletal anchorage, Xie et al^4^ presented the concept of bone-bonding anchorage, using an adhesive to attach the device to the bone. They demonstrated the fixation of a stainless steel piece to the cortical bone surface using N-butyl-2-cyanoacrylate adhesive. Synthetic cyanoacrylate adhesives are a family of liquid monomers that cure at room temperature, in an exothermic reaction on contact with a small amount of water or basic fluid. In 1988, the FDA approved 2-octyl-cyanoacrylate (Dermabond^®^) for topical use as a substitute for sutures, and, later, 2-butyl-cyanoacrylate (Hystoacryl^®^, Indermil^®^).^5^ ^,^ ^6^ These cyanoacrylate adhesives have been found to be biocompatible, bacteriostatic and hemostatic, in addition to offering easy handling^7^ and shorter operative time during surgical procedures.^8^

In an endeavor to overcome some of the disadvantages during mini-implant placement, the present group of authors developed a novel device with reduced dimensions and an active anchoring part in the shape of an arrow. The ‘Arrow’ device was designed to be placed in the cortical portion of the bone and facilitates its placement, enhancing mechanical anchorage. Furthermore, the use of a N-butyl-2-cyanoacrylate-based adhesive may result in better primary stability and a higher success rate. The ultimate purpose of this innovative concept is to create a skeletal anchorage system that ensures good stability and decreases the risk of damage to important structures during insertion. 

Therefore, using an animal model, the aims of this study were: to perform a histological assessment of the interlocking of the Arrow device to the cortical bone, by optical microscopy observation; to mechanically evaluate the maximum loading capacity by shear strength test; and to study the types of fractures that occurred during the mechanical test, by visual inspection immediately, 30 and 60 days after its insertion, with or without N-2-butyl-cyanoacrylate adhesive. 

## MATERIAL AND METHODS

This study was approved by the Ethics Committee on Animal Research of Federal University of Minas Gerais (protocol no. 330/2014).

**Figure 1:**
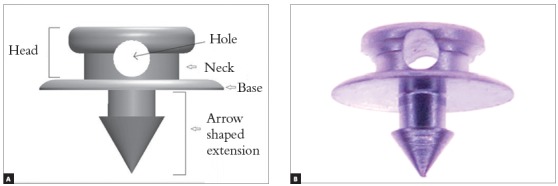
Arrow mini-implant: (A) Identification of device parts. (B) Mini-implant.

The device for orthodontic anchorage (Arrow) was made with commercially pure titanium grade 4 (ASTM F67) (PecLab, Belo Horizonte, Brazil). The Arrow is composed by three parts: head, base and an arrow-shaped extension. The head is 2.4 mm wide and 1.2 mm high, with a 0.7 mm diameter hole for orthodontic wire fixation (Fig 1). The base is 3.5 mm in diameter, with an arrow-shaped extension, 1.3 mm high and 1.25 mm wide. 

Ninety-six Arrow devices were placed bilaterally in the tibia of nine male New Zealand male white rabbits (similar to those used by Xie et al^4^), aged 5 to 6 months old and weighing 3 to 3.5 kg. This experimental design was based in that performed by Xie et al,^4^ to reduce the number of animals used in the study, as suggested by the Ethics Committee on Animal Research. From a total of 96 devices placed in the tibias, 90 were used to evaluate the shear strength, and the other six devices were used for histological evaluation. The factors studied were: (1) use or non-use of an adhesive for mini-implants fixation; (2) evaluation time points: immediately following, and 30 and 60 days after. Nine animals were divided into three groups. Twelve devices were placed bilaterally (six in each tibia) on one rabbit from each group, and ten devices (five in each tibia) were placed on the other animals. The devices were placed with adhesive on the right tibia of all animals, and without adhesive on the left tibia.

The surgical procedures began one week after the animals arrived at the laboratory. The animals were anesthetized with intramuscular ketamine (30 mg/kg) and xylazine (5 mg/kg). Following skin preparation, trichotomy and antisepsis with povidone-iodine, 2% lidocaine with epinephrine 1:100.000 was applied as the local anesthetic. All the surgeries were conducted under sterile conditions, in an operating room specific for animals.

**Figure 2:**
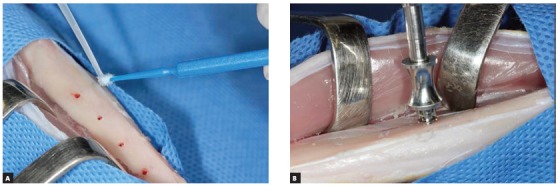
A) Adhesive application. B) Mini-implant placement.

The skin was incised and dissected, and the tibia was exposed. Five or six holes were made in the cortical bone with a 1.1 mm manual drill, at intervals of 8 mm. N-2-butyl-cyanoacrylate (Vetbond™, 3M, St. Paul, MN, USA) was used to place the mini-implants in the right tibia of the animals (Fig 2A). During insertion, the mini-implants were first fixed with a special locking device, and then a drop of the adhesive was dispensed onto an applicator brush, and immediately applied to the prepared surface. The Arrow devices were placed in position with a surgical hammer and a tweezer, under manual pressure, until the device was visually close to the bone surface (Fig 2B). No torque was applied during insertion of the devices. The soft tissues were closed with 4-0 nylon suture (Ethicon, São José dos Campos, SP, Brazil). The same procedure was used for the groups without adhesive, except for application of the adhesive. 

After surgery, the animals received analgesics and antibiotics intramuscularly every day for three consecutive days. According to the different group times (immediately following, and 30 and 60 days after), the animals were euthanized with an overdose of sodium pentobarbital 60-80 mg/kg IV. 

Bone sections 7-8 mm thick were removed from the animals that received one more device than the others in each tibia. The samples were fixed, embedded, sectioned and stained with Stevenel’s blue, Van Gieson’s and alizarin red. Histological analysis was performed by optical microscope, with a digital camera and with image processing software (AxioVision, Carl Zeiss, Gottingen, Germany).

**Figure 3:**
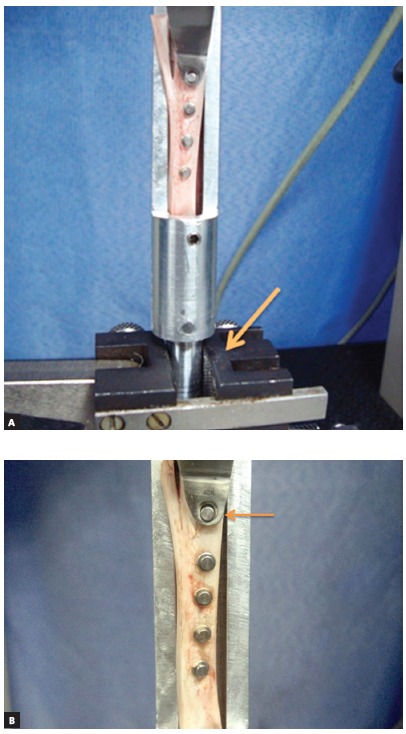
A) Metal device fixed in the machine. B) Locking device attached to the mini-implant.

The samples were submitted to shear strength testing on a universal testing machine (EMIC DL 2000, São José dos Pinhais, PR, Brazil), at a speed of 1 mm/min and a 50 kgf load cell. The test was performed by fixing a metal device, which held the tibia motionless inside the device, to the lower part of the machine (Fig 3A), and locking each mini-implant into place, by applying a horizontal force perpendicular to its major axis (Fig 3B).

Immediately following the shear strength test, visual inspection was performed by two operators, to evaluate the fracture types qualitatively: adhesive, when the fracture was observed in the contact surface between the mini-implant and the bone; cohesive in bone, when there was bone at the base of the implant; and cohesive in mini-implant, when there was a piece of mini-implant remaining in the bone. 

The statistical analysis was performed by verifying the assumptions of normality and homoscedasticity. The shear strength values of the mini-implants were submitted to analysis of variance (ANOVA) followed by Tukey’s test (*p*< 0.05), and the statistical analysis was performed using SPSS 20 (SPSS Inc., Chicago, IL, USA).

## RESULTS

All the 96 mini-implants inserted achieved primary stability. The rabbits had no complications during the healing process. Since all the mini-implants remained stable over the entire study, the success rate was 100%.

**Figure 4:**
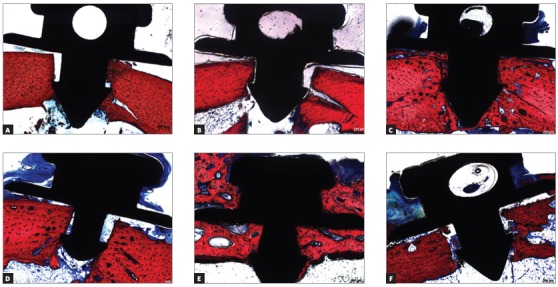
Photomicrographs of the mini-implant. (A) Immediate time point without adhesive. (B) Immediate time point with adhesive. (C) 30 days without adhesive. (D) 30 days with adhesive. (E) 60 days without adhesive. (F) 60 days with adhesive.

Figure 4 shows the histological results. In both groups, without (Fig 4A) and with adhesive (Fig 4B), the presence of gaps was observed at the immediate time point, between the mini-implant and the cortical bone, with no close contact between the surfaces.

After 30 days, the group without adhesive (Fig 4C) showed interlocking between the mini-implant and the cortical bone, and absence of gaps at this interface. In contrast, the group with adhesive (Fig 4D) showed gaps between the device and cortical bone, in the arrow-shaped extension and at the base of the device. 

After 60 days, the group without adhesive (Fig 4E) showed no gaps between the interface of the device and the cortical bone. However, gaps were observed in the group with adhesive (Fig 4F). In addition, the group without adhesive clearly showed that the cortical bone was practically wrapped around the device, from the arrow base to the head, whereas there was just one interlocking of the cortical bone lateral to the base, on the devices with adhesive.

The shear strength test produced no mini-implant fracture. The statistical analysis showed significant interaction between the ‘adhesive’ and the ‘time’ factors under study (*p*< 0.001). The Tukey test revealed no significant difference in shear strength of the mini-implants with or without use of cyanoacrylate, at the immediate time point. Nevertheless, after 30 and 60 days, the shear strength of the device was significantly higher in the absence of adhesive. When the cyanoacrylate was applied, the shear strength was not significantly affected by the time point (Table 1).

**Table 1:** Mean values and standard deviations of shear strength (Newtons) of Arrow anchorage, according to the use of cyanoacrylate adhesive and time points.

Time point	Cyanoacrylate adhesive to mini-implants fixation	
	Presence	Absence
Immediate	24.69 (12.27)^Aa^	20.70 (9.47)^Ac^
30 days	18.23 (11.50)^Ba^	43.98 (18.21)^Ab^
60 days	31.98 (14.19)^Ba^	78.55 (18.51)^Aa^

Means followed by uppercase letters indicate a significant difference between the presence and absence of adhesive, at each time point. Means identified by different lowercase letters show a significant difference among the times points, considering the use or non-use of adhesive.

**Figure 5:**
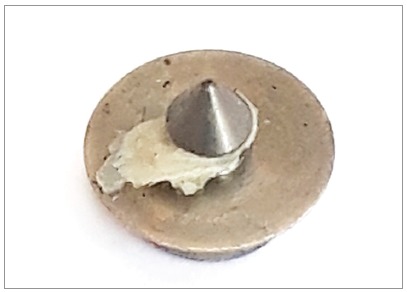
Mini-implant with cohesive in bone fracture.

Analyzing the types of fractures that occurred, adhesive failure corresponded to 100% at the immediate time point in the groups with and without adhesive, versus 75% at 30 days and 30% at 60 days in the group with adhesive. The cohesive in bone fractures corresponded to 100% in 30 and 60 days in the group without adhesive (Fig 5), *versus* 70% in 60 days and 25% in 30 days in the group with adhesive. There was no cohesive in mini-implant fracture (Table 2). 

**Table 2:** Distribution of fracture modes by study group.

Time point and adhesive	Fracture mode		
	Adhesive	Cohesive in bone	Cohesive in mini-implant
Immediate with adhesive	100%	0	0
Immediate without adhesive	100%	0	0
30 days with adhesive	75%	25%	0
30 days without adhesive	0	100%	0
60 days with adhesive	30%	70%	0
60 days without adhesive	0	100%	0

## DISCUSSION

The size of the mini-implant is a factor that may hinder its use in certain areas and increase the risk of damage to adjacent structures,^3^ ^,^ ^9^ whereas the proximity of the mini-implant to the root can lead to loss of the device,^10^ root resorption and ankylosis. It has been suggested that mini-implants should be small enough to be placed anywhere in the alveolar bone.^3^

The Arrow was developed for the aforementioned reasons. The structure of this novel device is arrow-shaped at its active extremity, and offers conicity and more manageable dimensions, good primary stability and low surgical trauma during insertion.^11^ The device is placed in such a way that its active part is located almost entirety in the cortical bone due to its dimensions^12,13^ (Fig 4), thereby reducing the risk of perforating the dental root or other important anatomical structures. Because of the reduced dimensions of its design, Arrow was manufactured with commercially pure titanium Grade 4, to enable osseointegration. As shown in the present study, this novel mini-implant did not fracture during the shear strength test performed at different time points, despite the finding that osseointegration may increase the possibility of mini-implant fracture.^14^ Although the reduced length of the device may contribute to fracture resistance, as observed by Vilani et al,^15^ who reported that shorter mini-implants have a lower risk of fracture, this occurrence was not reported in the present study. The absence of fractures may be related to the mechanical properties of the titanium Grade 4, which has high strength, with minimum yield strength of 480 MPa. This alloy combines excellent corrosion resistance and good formability and weldability.^16^

 In the present study, 100% of the devices showed osseointegration and cohesive bone fracture after 30 and 60 days in the group without adhesive. In both the adhesive and non-adhesive groups, there was adhesive failure only at the immediate time point, since there was no time for osseointegration. Although Arrow design could provide immediate stability and provided osseointegration over time, cohesive bone fracture is related as one of the limitations of this anchorage device, and methods to minimize bone loss during removal of this device need to be developed. Furthermore, it was observed that the use of adhesive led to a lower prevalence of cohesive bone fractures (25% and 70% in 30 and 60 days, respectively), and that the presence of the adhesive hindered osseointegration. 

Analyzing images of the histological sections of the group without adhesive at the immediate time point, gaps were observed between the bone and the device (Fig 4A), since there was no time for osseointegration. At the 30- and 60-day time points (Fig 4C and 4E), interlocking of newly formed cortical bone with mini-implant was observed without gaps, showing osseointegration. On the other hand, gaps were noticed between the newly formed cortical bone and the device, along the arrow-shaped extension (Fig 4B, 4D and 4F) in the group with adhesive, at all the time points. This is probably due to the adhesive present on these sites, as also observed by Xie et al.^4^

As shown in Table 1, there was no significant difference in shear strength between the groups with or without adhesive, at the immediate time point. However, it should be considered that the application of adhesive could have some effect on mini-implant to bone adherence. This could explain the tendency of higher average load values (24.69 N) of the adhesive *versus* non-adhesive group (20.70 N). All the groups with adhesive showed no significant difference in shear strength average values, and trended toward decreasing values at 30 days (18.23 N) and increasing values at 60 days (31.98 N). The shear strength of the device was significantly higher in the absence of adhesive (43.98 N at 30 days, and 78.55 N at 60 days), compared with the adhesive group. The lower average values observed in the adhesive group may be related to the cyanoacrylate not being metabolized,^17^ thereby hindering the interlocking of newly formed bone with the titanium device.^18^

A systematic review^19^ reported that the shear strength levels used in orthodontic treatments ranged from 50 to 400 g, but the majority of studies have used 200 g or less.^20^ Once rigid fixation (secondary stability) is achieved, orthodontic forces are not a threat to mini-implants and bone integration.^21^ In this study, mechanical evaluation conducted with the shear strength test indicated that all the groups with and without adhesive achieved average values above the optimum values required for use in orthodontic skeletal anchorage, at all the tested time points.

Comparing the Arrow device used in the adhesive group with the device used by Xie et al,^4^ the latter had lower values (10.84 N) at the immediate time point, and also exhibited a decreasing trend in average values at 15 days (6.23 N) and 30 days (1.8 N), and an increasing trend at 60 days (45.69 N), all indicating a significant difference. According to these authors, the decreasing trend occurred because of reduced adhesion strength, due to adhesive hydrolysis. In the adhesive group, higher average shear strength values were verified at the immediate time point and at 30 days, compared with those by Xie et al^4^ at the same time points. This is probably due to the arrow-shaped structure extending from the base, which anchors the device and keeps it in position. Regarding the results observed after 60 days, Xie et al^4^ recorded higher values than the present study at this same time point, using a device with a flat base, probably due to the amount of new bone tissue around the device. 

The thickness of the cortical bone has a major impact on primary stability, especially in dolichofacial patients, with high mandibular and gonial angles, because their cortical bone is very thin.^11,12,13^ Alternative anchorage devices should be used on these patients to achieve primary stability. Other considerations for these devices include the use of biological adhesive, a greater device diameter, a surface treatment or the combined insertion of mini-implant and auxiliary accessories with indentations facing the cortical bone.^3^ ^,^ ^12^ ^,^ ^22^ The application of small amounts of the adhesive lateral to the device^23^ should be evaluated for primary stability in patients with thin cortical bone. 

Better results for the mini-implants, especially in patients with thin cortical bone, could be obtained by developing an adhesive agent, together with a method for its application, which ensures primary stability in almost 100% of the cases, thus promoting an increase in bone implant contact, achieving secondary stability, so that it can be used as an orthodontic anchorage. Further studies are needed to assess the best location of the biological adhesive during device insertion, use of other types of adhesive, a shear strength test that simulates orthodontic active treatment, and a way of removing the device in a clinical trial.

## CONCLUSIONS

There was new bone formation, achieving close contact with the device in 30-60 days in the nonadhesive group. In the adhesive group, there were gaps between the device and the bone. At the immediate time point, the shear strength was similar between the groups with and without adhesive. Higher shear strength was found for the group without adhesive at 30 and 60 days, increasing over time. The Arrow showed no mini-implant fracture. 

## References

[1] Melsen B (2011). Miniscrew loosening. J Clin Orthod.

[2] Holmes PB, Wolf BJ, Zhou J (2015). A CBCT atlas of buccal cortical bone thickness in interradicular spaces. Angle Orthod.

[3] Lee YK, Kim JW, Baek SH, Kim TW, Chang YI (2010). Root and bone response to the proximity of a mini-implant under orthodontic loading. Angle Orthod.

[4] Xie X, Bai Y, Lv Y, Gao W (2010). A study on orthodontic bone-bonding anchorage. Angle Orthod.

[5] Mehdizadeh M, Yang J (2013). Design strategies and applications of tissue bioadhesives. Macromol Biosci.

[6] Sanders L, Nagatomi J (2014). Clinical applications of surgical adhesives and sealants. Crit Rev Biomed Eng.

[7] Saska S, Hochuli-Vieira E, Minarelli-Gaspar AM, Gabrielli MF, Capela MV, Gabrielli MA (2009). Fixation of autogenous bone grafts with ethyl-cyanoacrylate glue or titanium screws in the calvaria of rabbits. Int J Oral Maxillofac Surg.

[8] Brown PN, McGuff HS, Noorily AD (1996). Comparison of N-octyl-cyanoacrylate vs suture in the stabilization of cartilage grafts. Arch Otolaryngol Head Neck Surg.

[9] Kuroda S, Yamada K, Deguchi T, Hashimoto T, Kyung HM, Takano-Yamamoto T (2007). Root proximity is a major factor for screw failure in orthodontic anchorage. Am J Orthod Dentofacial Orthop.

[10] Miyawaki S, Tomonari H, Yagi T, Kuninori T, Oga Y, Kikuchie M (2015). Development of a novel spike-like auxiliary skeletal anchorage device to enhance miniscrew stability. Am J Orthod Dentofacial Orthop.

[11] Wilmes B, Rademacher C, Olthoff G, Drescher D (2006). Parameters affecting primary stability of orthodontics mini-implants. J Orofac Orthop.

[12] Holmes PB, Wolf BJ, Zhou J (2015). A CBCT atlas of buccal cortical bone thickness in interradicular spaces. Angle Orthod.

[13] Veli I, Uysal T, Baysal A, Karadede I (2014). Buccal cortical bone thickness at miniscrew placement sites in patients with different vertical skeletal patterns. J Orofac Orthop.

[14] Consolaro A, Romano FL (2014). Reasons for mini-implants failure: choosing installation site should be valued. Dental Press J Orthod.

[15] Vilani GNL, Ruelas ACO, Mattos CT, Fernandes DJ, Elias CN (2015). Influence of cortical thickness on the stability of mini-implants with microthreads. Braz Oral Res.

[16] Islamgalieva RK, Kazyhanova VU, Shestakovaa LO, Sharafutdinovb AV, Valieva RZ (2008). Microstructure and mechanical properties of titanium (Grade 4) processed by high-pressure torsion. Mater Sci Eng.

[17] Xavier MSV, Leite VM (2012). The effect of 2-butyl-cyanoacrylate adhesive in osteotomies and bone grafts in rabbits macroscopic and radiographic characteristics. Rev Bras Ortop.

[18] Esteves JC, Borrasca AG, Arenega AM, Garcia IR, Magro O (2011). Histomorphometric analysis of the repair process of autogenous bone grafts fixed at rat calvaria with cyanoacrylate. J Appl Oral Sci.

[19] Reynders R, Ronchi L, Bipat S (2009). Mini-implants in orthodontics: A systematic review of the literature. Am J Orthod Dentofacial Orthop.

[20] Zhang L, Zhao Z, Li Y, Wu J, Zheng L, Tang T (2010). Osseointegration of orthodontic micro-screws after immediate and early loading. Angle Orthod.

[21] Vannet BV, Sabzevar MM, Wehrbein H, Asscherickx K (2007). Osseointegration of miniscrews a histomorphometric evaluation. Eur J Orthod.

[22] Tozlu M, Nalbantgil D, Ozdemir F (2013). Effects of a newly designed apparatus on orthodontic skeletal anchorage. Eur J Dent.

[23] Bas B, Ozden B, Bekçioglu B, Sanal KO, Gülbahar MY, Kabak YB (2012). Screw fixation is superior to N-butyl-2-cyanoacrylate in onlay grafting procedure a histomorphologic study. Int J Oral Maxillofac Surg.

